# Efficacy, safety and the lymphocyte subsets changes of low‐dose IL‐2 in patients with systemic lupus erythematosus: A systematic review and meta‐analysis

**DOI:** 10.1002/iid3.1165

**Published:** 2024-01-24

**Authors:** Qin‐Yi Su, Jing Luo, Xin‐Miao Wang, Jing‐Kai Di, Yi‐Xin Cao, Sheng‐Xiao Zhang

**Affiliations:** ^1^ Department of Rheumatology The Second Hospital of Shanxi Medical University Taiyuan Shanxi China; ^2^ Shanxi Provincial Key Laboratory of Rheumatism Immune Microecology Taiyuan Shanxi China; ^3^ Key Laboratory of Cellular Physiology at Shanxi Medical University Ministry of Education Taiyuan China

**Keywords:** interleukin‐2, lupus erythematosus, regulatory, systemic, T‐lymphocytes, Th17 cells

## Abstract

**Introduction:**

Existing therapies of systemic lupus erythematosus (SLE) are efficacious only in certain patients. Developing new treatment methods is urgent. This meta‐analysis aimed to evaluate the efficacy and safety of low‐dose IL‐2 (LD‐IL‐2).

**Methods:**

According to published data from PubMed, Web of Science, Embase, ClinicalTrials.gov, MEDLINE, MEDLINE, Web of Knowledge, Cochrane Library, and FDA.gov, eight trials were included.

**Results:**

After the LD‐IL‐2 treatment, 54.8% of patients had distinct clinical remission. The SRI‐4 response rates were 0.819 (95% confidence interval [CI]: 0.745–0.894), and the SELENA‐SLEDAI scores were significantly decreased (SMD = −2.109, 95% CI: [−3.271, −0.947], *p* < .001). Besides, the proportions of CD4^+^ T (SMD = 0.614, 95% CI: [0.250, 0.979], *p* = .001) and Treg cells (SMD = 1.096, 95% CI: [0.544, 1.649], *p* < .001) were increased dramatically after LD‐IL‐2 treatment, while there were no statistical differences in the proportions of CD8^+^ T cells, Th1 cells, Th2 cells, and Th17 cells (*p* > .05). Besides, the proportions of Th17 (SMD = 1.121, 95% CI: [0.709, 1.533], *p* < .001) and Treg (SMD = 0.655, 95% CI: [0.273, 1.038], *p* = .001) were significantly increased after receiving subcutaneously 0.5 million IU of LD‐IL‐2 treatment per day for 5 days, but there were no statistical differences in the proportions of Treg after receiving 1 million IU every other day subcutaneously of LD‐IL‐2 treatment. Injection site reaction and fever were common side effects of IL‐2, which occurred in 33.1% and 14.4% of patients. No serious adverse events were reported.

**Conclusion:**

LD‐IL‐2 was promising and well‐tolerated in treating SLE, which could promote Treg's proliferation and functional recovery. Injecting 0.5 million IU of IL‐2 daily can better induce the differentiation of Treg cells and maintain immune homeostasis than injecting 1 million IU every other day.

## Graphical abstract

Low‐dose IL‐2 was promising and well‐tolerated in treating systemic lupus erythematosus, which could promote Treg's proliferation and functional recovery. Injecting 0.5 million IU of IL‐2 daily can better induce the differentiation of Treg cells and maintain immune homeostasis than injecting 1 million IU every other day.

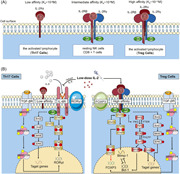

## 1 INTRODUCTION

Systemic lupus erythematosus (SLE) is an autoimmune disease with a wide range of manifestations involving one or more organs, such as the skin, kidneys, joints, and nervous system.^1^ With high prevalence, extremely harmful effects, and the unavailability of effective medical treatments, SLE has become a severe threat to human health. Clinicians use a broad range of drugs to treat SLE, mainly relying on glucocorticoids, antimalarial agents, nonsteroidal anti‐inflammatory drugs (NSAIDs), immunosuppressive agents, and B‐cell‐targeting biologics, but these drugs are efficacious only in certain patients. Therefore, developing new treatment methods is urgent.^2^, ^3^, ^4^

Interleukin‐2 (IL‐2) is a central cytokine that can dose‐dependently promote the expansion and differentiation of different immune cell subsets. At low doses, IL‐2 promotes Tregs, while at high doses, it promotes effector cells such as CD8^+^ T cells and natural killer (NK) cells.^5^ A disruption of Tregs homeostasis caused by an acquired deficiency of IL‐2 is a crucial event in the pathogenesis of SLE. It propagates the immune response and terminates it by promoting the activation‐induced cell death of T cells.^6^, ^7^ Treatment with low‐dose IL‐2 (LD‐IL‐2) selectively expands Tregs, thereby this approach has been explored as a new clinical strategy for SLE.

Although clinical trials suggested that LD‐IL‐2 therapy is capable of promoting the selective expansion of a functionally competent Treg population in a well‐tolerated way and may have the potential to influence the clinical course in patients with active SLE, evidence‐based medicine evidence is inadequate, with low sample sizes and limited elaboration, no systematic data to comprehensively explain its safety, effectiveness and the optimal dosage of LD‐IL‐2 (1 million IU every other day or 0.5 million IU per day).^8^, ^9^ In this study, we aimed to evaluate the efficacy and safety of LD‐IL‐2 and the effect of LD‐IL‐2 on Treg status and immune balance.

## 2 MATERIALS AND METHODS

### 2.1 Data sources and search strategy

This systematic meta‐analysis was conducted by the Preferred Reporting Items for Systematic Reviews and Meta‐Analyses (PRISMA) reporting guidelines and registered on the International Prospective Register of Systematic Reviews (PROSPERO) trial registry (CRD42021290139).^10^

We searched for relevant studies published from inception to December 10, 2023, on PubMed, EMBASE, Web of Science, the Cochrane Library and Medline, CNKI, CBM, and Technology Journal Database, with no restrictions on publication language or primary outcome. The articles were retrieved using a combination of subject terms and free words. The relevant papers were identified using Medical Subject Headings (MeSH) terms: “Interleukin‐2,” “Lupus Erythematosus, Systemic,” and related free words. In addition, we manually searched the reference lists of published systematic reviews and original articles.

### 2.2 Study selection and data extraction

Inclusion criteria were observational studies (prospective and retrospective), and clinical trials that reported efficacy or safety data in patients with SLE receiving IL‐2 therapy. The meta‐analysis of the results in original studies conducted on humans with the following MeSH terms and free words in the title or abstract “Lupus Erythematosus, Systemic” and “Interleukin‐2.” The included studies must measure the levels of SLE Responder Index‐4 (SRI‐4) and Safety of Estrogens in Lupus Erythematosus National Assessment version of the SLE Disease Activity Index (SELENA–SLEDAI), adverse events, Treg cells, Th17 cells, Th1 cells, Th2 cells, CD4^+^ cells, CD8^+^ cells, or NK cells in patients with SLE before and after treatment, with no limitation on the disability level, age, race/ethnicity, sex of study participants or the time of publication.

The meta‐analysis excluded nonoriginal studies and multiple reports of the same or overlapping data. In addition, we excluded studies with missing data that could not be obtained even after contacting the authors.

Two investigators independently assessed the studies based on the eligibility criteria; disagreements were resolved by a third investigator. A data collection sheet was used to record the first author's name, publication year, sample size, disease duration, treatment regimen, exposure duration, concomitant therapy, history of prior treatment, efficacy measures, and diagnostic criteria. Evidence was graded according to the Oxford Centre for Evidence‐based Medicine 2011 Levels of Evidence. The quality of studies was assessed using the Newcastle‐Ottawa quality assessment scale (NOS) (Table 1).^20^

**Table 1:** The baseline of patients with SLE included in the study.

Study (year)	No. of patient	*Q*	Dynamics of immune cell subsets		SELENA–SLEDAI
			Th17 cells, mean ± SD	Tregs, mean ± SD	
He et al. (2019)^8^	30	7		Before: 12.77 ± 10.25 cells/μL; After: 14.19 ± 6.07 cells/μL	Before: 12 ± 4.75; After: 6 ± 4
He et al. (2016)^11^	38	6	Before: 4.1 ± 1.28 cells/μL; After: 2.67 ± 2.07 cells/μL	Before: 10.64 ± 3.67 cells/μL; After: 14.11 ± 4.03 cells/μL	Before: 11.14 ± 3.79; After: 3.98 ± 2.23
Shao et al. (2019)^12^	15	7		Before: 7.24 ± 5.21 cells/μL; After: 10.19 ± 5.69 cells/μL	
Zhao et al. (2019)^13^	50	7		Before: 16.9 ± 1.69 cells/μL; After: 32.73 ± 8.27 cells/μL	Before: 5.92 ± 0.36; After: 4.05 ± 0.31
Zhang et al. (2019)^14^	54	8	Before: 7.42 ± 0.62 cells/μL; After: 10.79 ± 10.67 cells/μL	Before: 34.02 ± 1.24 cells/μL; After: 57.55 ± 45.19 cells/μL	
Wang et al. (2017)^15^	76	7	Before: 6.29 ± 6.01 cells/μL; After: 10.02 ± 9.19 cells/μL	Before: 14.87 ± 11.4 cells/μL; After: 56.69 ± 40.76 cells/μL	Before: 10.87 ± 6.48; After: 5.48 ± 4.13
Zhang et al. (2022)^16^	41	8	Before: 8.5 ± 6.6 cells/μL; After: 15.3 ± 21.4 cells/μL	Before: 28 ± 33 cells/μL; After: 80 ± 50 cells/μL	
Humrich et al. (2022)^17^	24	5			Before: 11.3 ± 3.37; After: 5.18 ± 1.75
Zhou et al. (2023)^18^	11	6			Before: 9.72 ± 5.26; After: 8.26 ± 3.36
Fan et al. (2018)^19^	76	6			

*Note*: Values are mean ± SD.

Abbreviation: SELENA–SLEDAI, Safety of Estrogens in Lupus Erythematosus National Assessment version of the SLE Disease Activity Index.

### 2.3 Statistical analysis

The SRI‐4 and the SELENA‐SLEDAI are important indicators for evaluating disease activity of SLE. Disorder of immune cell subsets especially Tregs and Th17 cells reflected severity of SLE. Based on the variable clinical presentations and heterogeneous definitions of treatment response in the included studies, we assessed the effect of IL‐2 treatment on two outcomes: the efficacy assessed by the primary and secondary endpoints. The primary endpoint was the SRI‐4 at Week 12 and the SELENA–SLEDAI. The secondary endpoint was the dynamics of immune cell subsets, such as the levels of Treg cells, Th17 cells, Th1 cells, Th2 cells, CD4^+^ cells, CD8^+^ cells, or NK cells. Flow cytometry was used for enumeration of lymphocyte subsets. Safety was assessed on the basis of the incidence of adverse drug reactions (ADRs) and severe adverse reactions.

We used Stata 12.0 software (Stata Corp.) for the meta‐analysis. The pooled efficacy (increase in SRI‐4 and reduction in SLEDAI) was calculated using random effects. In terms of safety profile, the occurrence of each adverse event was recorded or computed when available. Standardized mean difference (SMD) to the distribution and 95% confidence interval (CI) were used to assess the levels of Treg cells, Th17 cells, Th1 cells, Th2 cells, CD4^+^ cells, CD8^+^ cells, or NK cells in SLE. The *I*‐squared value (*I* ^2^) was used to assess the statistical heterogeneity between studies. An *I* ^2^ value of <25% indicates low heterogeneity, 25%–75% as moderate heterogeneity, and >75% as considerable heterogeneity. In case of significant heterogeneity (*I* ^2^ > 50%), the analysis was conducted under the random‐effects model. Otherwise, a fixed‐effect model was used. Subgroup analysis was performed in the patients treated with different regimens to handle potential heterogeneity. All patients with SLE involved in our study received conventional therapy including glucocorticoids and common csDMARDs, such as methotrexate (MTX), leflunomide (LEF), sulfasalazine (SSZ), and antimalarials such as hydroxychloroquine (HCQ) before IL‐2 injection. Data was explained individually when LD‐IL‐2 combined with other drugs.

Sensitivity analysis was assessed by separately leaving each study out and rerunning the analysis with data. Funnel plots and Egger's regression test were utilized to estimate the publication bias. Sensitivity analysis and Egger's and Begg's tests were conducted by STATA 12.0 software. We consider the result did not identify publication bias when a *p* > .05 in Egger's test.

## 3 RESULT

### 3.1 Results of the search strategy

**Figure 1:**
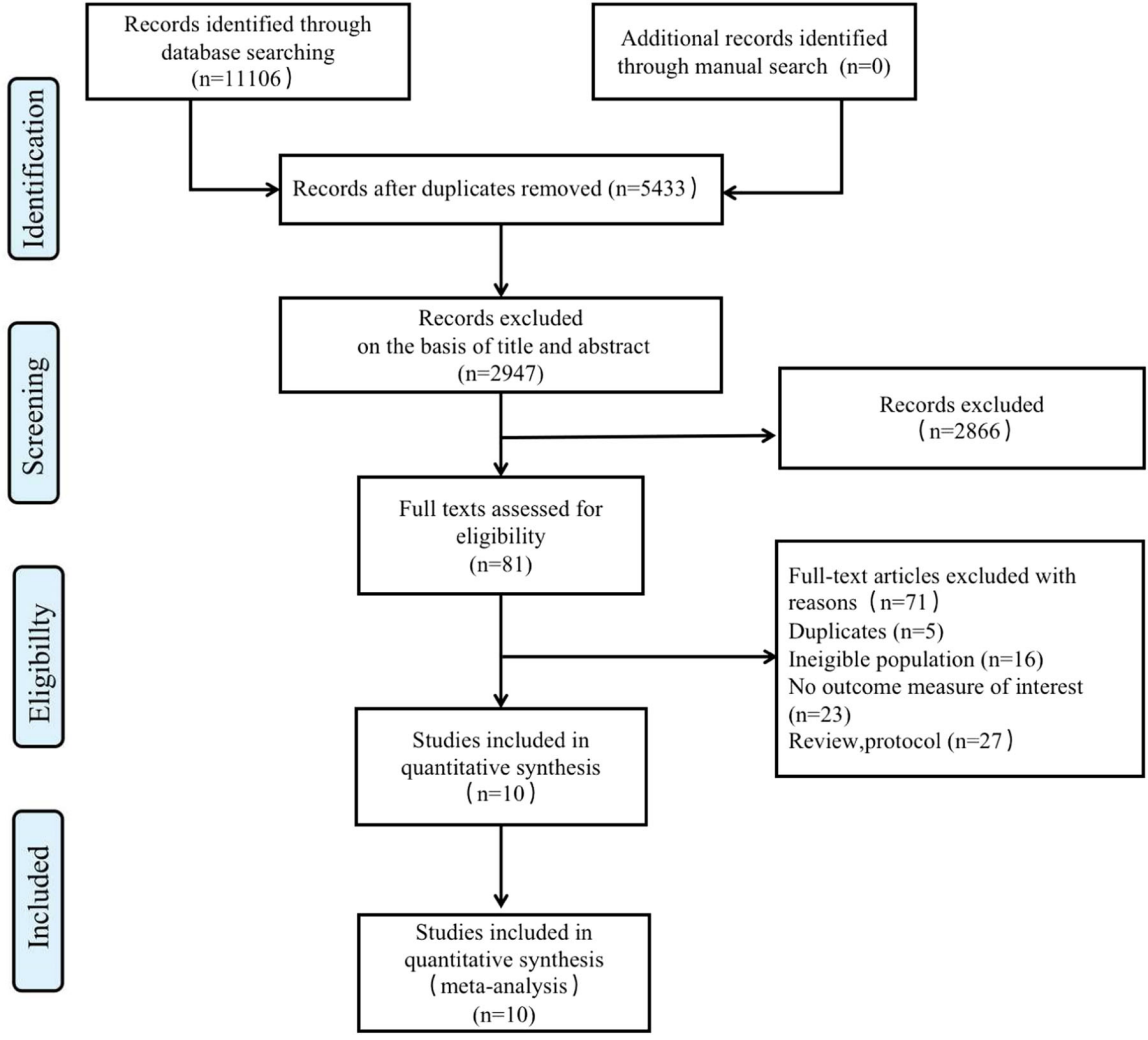
Flow chart of the assessment of the studies identified in the meta‐analysis.

We identified 11,106 citations through the literature search, a total of 3209 articles were retrieved from PubMed, 3361 articles were retrieved from EMBASE, 1983 articles were retrieved from Web of Science, 315 articles were retrieved from the Cochrane Library and Medline, 920 articles were retrieved from CNKI, 526 articles were retrieved from CBM and 792 articles were retrieved from Technology Journal Database. A total of 2866 titles and abstracts were excluded after initial screening. Based on exclusion criteria for full‐text review, which included duplicates, ineligible population, no outcome measure of interest and review and protocol, we assessed 10 studies for eligibility. Ineligible population included population lost to follow‐up and combined with other serious diseases, such as severe adult congenital heart disease, severe insulin‐resistant and insulin‐deficient diabetes. The final 10 full‐text articles met all eligibility criteria (Figure 1). These eight included studies were published between 2016 and 2023 and comprised 415 patients. The mean age of included patients was 18–65 years, with female predominance (82.5%–100%). The median disease duration was 66.7–103.7 months.

### 3.2 Efficacy of LD‐IL‐2 in patients with SLE

#### 3.2.1 Primary efficacy outcomes

**Figure 2:**
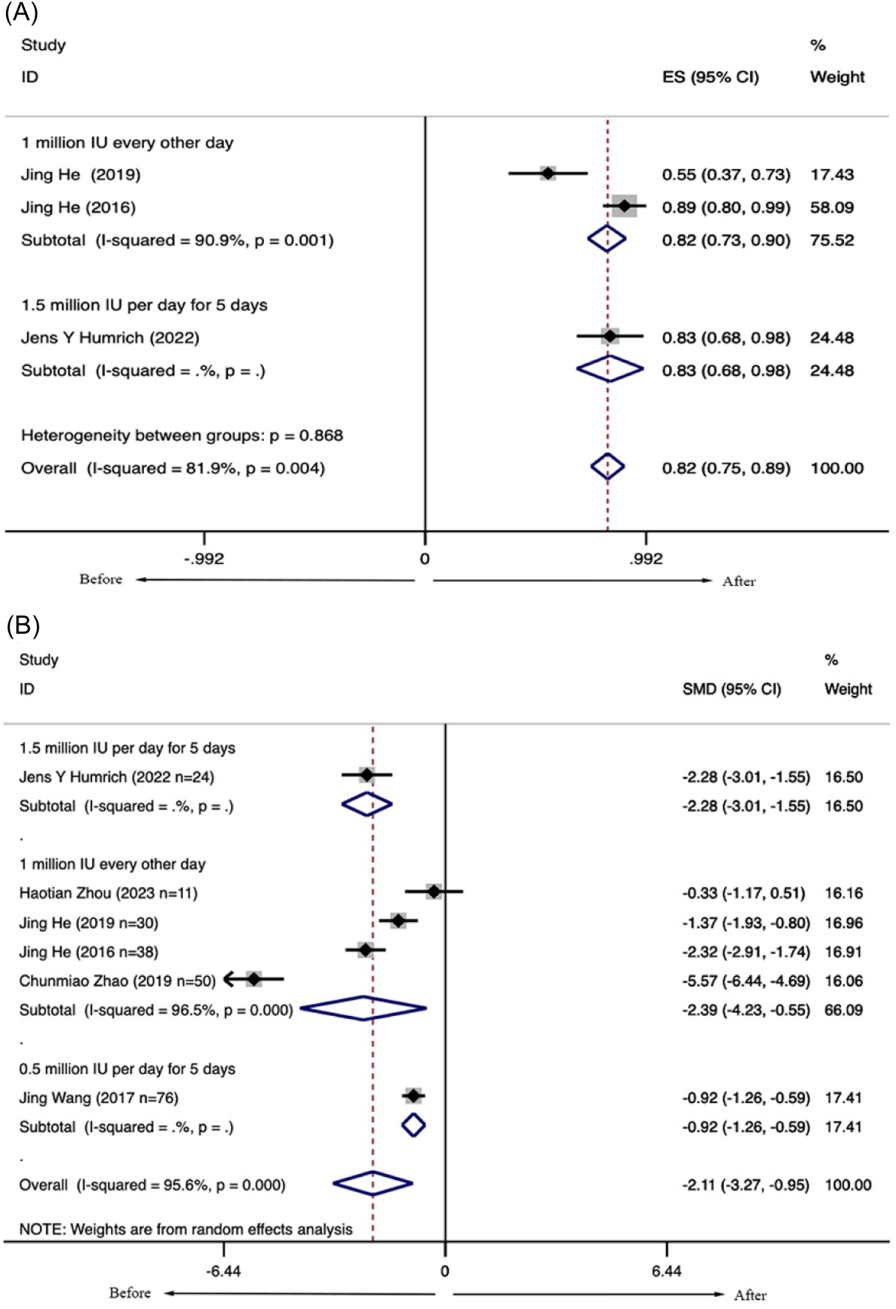
(A) The SRI‐4 response rates of systemic lupus erythematosus (SLE) treated with low‐dose interleukin‐2 (LD‐IL‐2) after 12 weeks. (B) The Safety of Estrogens in Lupus Erythematosus National Assessment version of the SLE Disease Activity Index scores of SLE were treated with LD‐IL‐2 after 12 weeks.

SRI‐4 response is defined as (1) a ≥4‐pointreduction in SELENA–SLEDAI score; (2) no new British Isles Lupus Assessment Group 2004 (BILAG) A (representing very active disease) score or ≤1 new BILAG B (representing moderate active disease) score; and (3) no deterioration from baseline in the physician's global assessment by ≥0.3 points. Meta‐analysis of three observational studies, including 92 patients with SLE, showed that the SRI‐4 response rate of patients treated with LD‐IL‐2 after 12 weeks was 0.819 (95% confidence interval [CI]: 0.745–0.894) (Figure 2A). These patients received LD‐IL‐2 1 million IU every other day for 2 weeks (a total of seven doses), followed by a 2‐week break or received LD‐IL‐2 1.5 million IU every day for 5 days followed by weekly injections for 12 weeks. There was statistically significant heterogeneity (*I* ^2^ = 81.9%). According to high heterogeneitywe performed a subgroup analysis (1.5 million IU per day: [SMD = 0.830, 95% CI: [0.680, 0.981], *p* < .001]); 1 million IU per day: (SMD = 0.816, 95% CI: [0.730, 0.901], *p* < .001]). But the heterogeneity was still high, it may be due to the difference between patients with SLE in age, gender, age at disease onset, disease duration, and treatment.

Meta‐analysis of six observational studies, including 229 patients with SLE, showed that the SELENA‐SLEDAI scores were significantly decreased (SMD = −2.109, 95% CI: [−3.271, −0.947], *p* < .001). The Egger test revealed no obvious publication bias (*t* = −1.490, *p* = .211). Considering the effect of different methods of dosing on the treatment effect, we performed a subgroup analysis. Patients received 1 million IU of LD‐IL‐2 every other day for 2 weeks, followed by a 2‐week break, and measured SELENA‐SLEDAI after 12 weeks. The SELENA‐SLEDAI scores were significantly lower than before treatment (SMD = −2.386, 95% CI: [−4.225, −0.546], *p* < .001) (Figure 2B).

Neither funnel plots (Figure S1) nor Egger tests showed evidence of publication bias in included studies of the SELENA‐SLEDAI scores (*t* = −1.49, *p* = .211).

Sensitivity analysis was to examine whether the SELENA‐SLEDAI scores were interfered by individual studies. There was no significant influence on the pooled results when any individual study was removed (Figure S4).

#### 3.2.2 Secondary efficacy outcome

**Figure 3:**
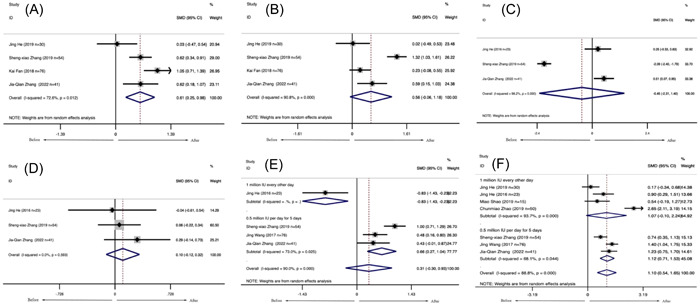
The proportion of lymphocyte subsets in patients with systemic lupus erythematosus after interleukin‐2 injection. (A) CD4^+^, (B) CD8^+^, (C) Th1, (D) Th2, (E) Th17, and (F) Treg.

**Figure 4:**
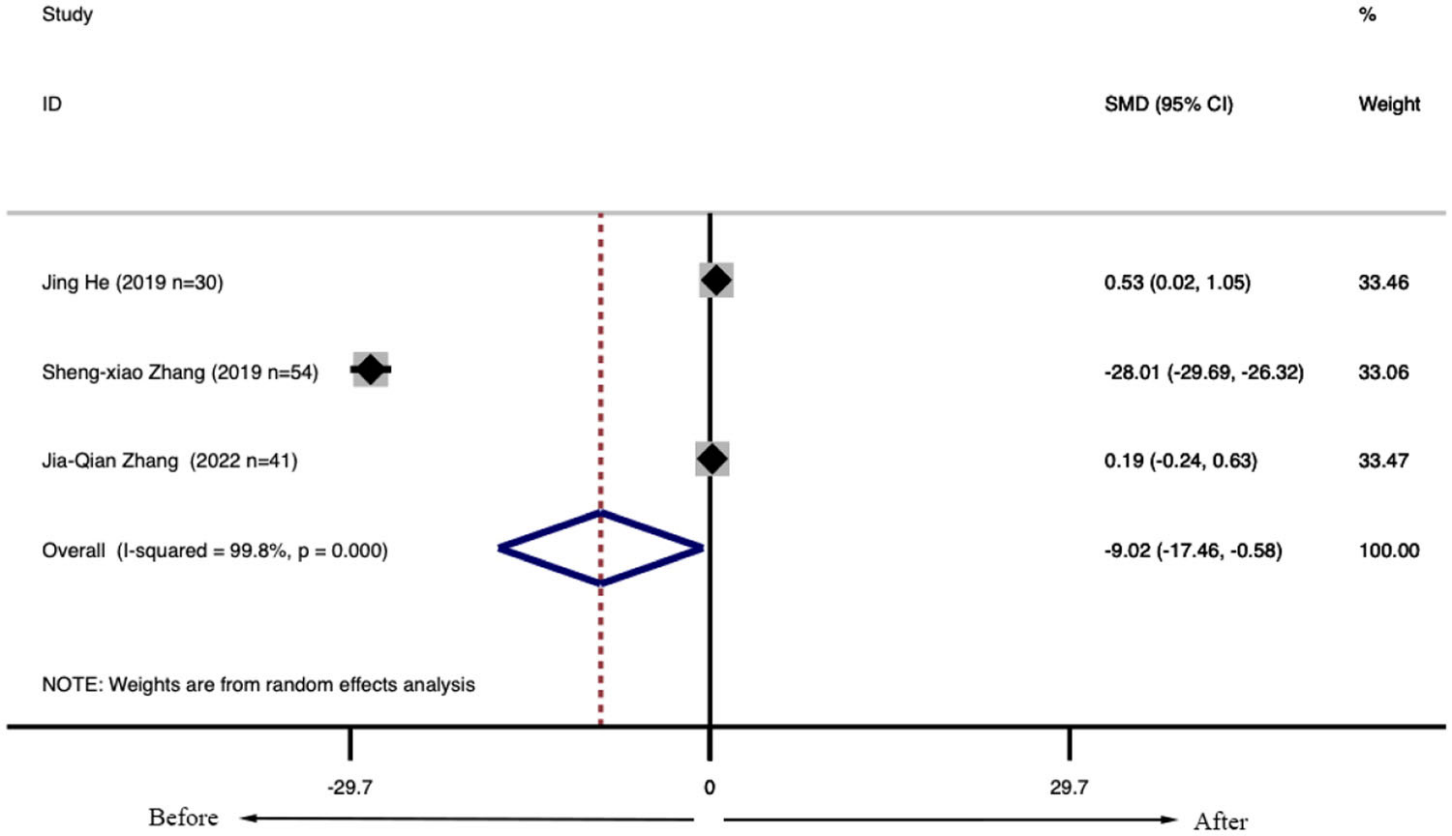
The proportion of NK cells in patients with systemic lupus erythematosus after interleukin‐2 injection.

Eight studies, including 380 patients with SLE, reported the levels of lymphocyte subsets in SLE treated with LD‐IL‐2. The meta‐analysis of the proportion of Treg cells showed that patients with SLE had a higher proportion of Treg cells after receiving LD‐IL‐2 compared with these patients before treatment (SMD = 1.096, 95% CI: [0.544, 1.649], *p* < .001) (Figure 3F), while there were no statistical differences in the proportions of Th17 cells (SMD = 0.315, 95% CI: [−0.297, 0.926], *p* = .313) (Figure 3E), Th1 cells (SMD = −0.454, 95% CI: (−2.309, 1.400), *p* < .001) (Figure 3C) and Th2 cells (SMD = 0.102, 95% CI: (−0.116, 0.321), *p* = .358) (Figure 3D) before and after LD‐IL‐2 treatment. The proportions of NK cells (CD56) were significantly decreased after LD‐IL‐2 injection (SMD = −9.019, 95% CI: [−17.460, −0.577], *p* = .036) (Figure 4). Besides, the proportions of CD4^+^ T cells were significantly increased after IL‐2 injection (SMD = 0.614, 95% CI: [0.250, 0.979], *p* = .001) (Figure 3A). At the same time, there were no statistical differences in the proportions of CD8^+^ T cells (SMD = 0.555, 95% CI: (−0.064, 1.175), *p* = .079) (Figure 3B) before and after IL‐2 treatment.

The subgroup analysis based on different dosages of LD‐IL‐2 showed no statistical differences in the proportions of Treg cells after receiving 1 million IU of LD‐IL‐2 every other day. For patients receiving 0.5 million IU per day of IL‐2 injection for 5 days, the proportions of Treg cells were significantly increased (SMD = 0.655, 95% CI: [0.273, 1.038], *p* = .001) (Figure 3E) and the proportions of Th17 cells were transiently increased (SMD = 1.121, 95% CI: (0.709, 1.533), *p* < .001) (Figure 3E). Heterogeneity was considerable overall (*I* ^2^ = 88.8%) and subgroup analyses (*I* ^2^ = 68.1%–93.7%).

Neither funnel plots (Figures S2 and S3) nor Egger tests showed evidence of publication bias in included studies of Th17 cells (*t* = −3.38, *p* = .078), Treg cells (*t* = −0.19, *p* = .859), Th1 cells (*t* = 1.75, *p* = .331), Th2 cells (*t* = 0.12, *p* = .927), CD4^+^ cells (*t* = −0.91, *p* = .458), CD8^+^ cells (*t* = −1.04, *p* = .408), NK cells (*t* = −4.95, *p* = .127).

Sensitivity analysis was to examine whether CD4^+^ cells, CD8^+^ cells, NK cells, Th1 cells, Th2 cells, Th17 cells, Treg cells were interfered by individual studies. The results of Th1 cells and NK cells were not robust. There was no significant influence on the pooled results in CD4^+^ cells, CD8^+^ cells, Th2 cells, Th17 cells, Treg cells when any individual study was removed (Figures S5 and S6).

#### 3.2.3 Efficacy of LD‐IL‐2 in lupus nephritis

**Figure 5:**
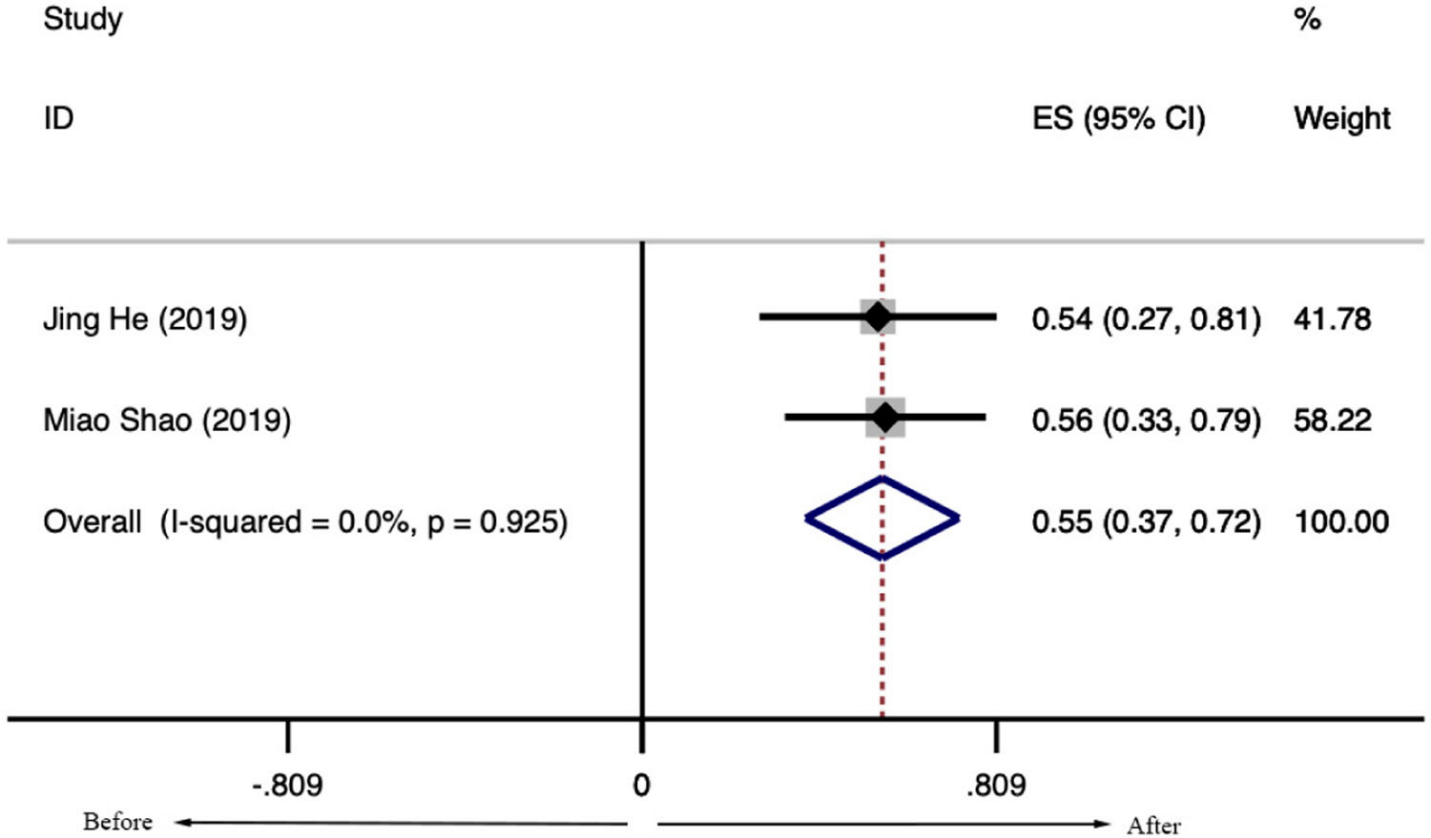
The remission rate of lupus nephritis patients after the low‐dose interleukin‐2 treatment.

After the LD‐IL‐2 treatment, 54.8% of lupus nephritis patients had distinct clinical remission (Figure 5).

### 3.3 Safety of LD‐IL‐2 treatment in patients with SLE

**Figure 6:**
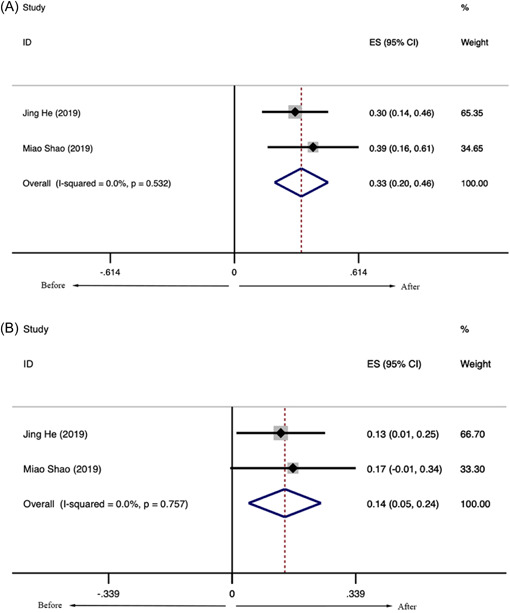
Incidence of adverse events in patients with systemic lupus erythematosus after the low‐dose interleukin‐2 treatment. (A) Injection site reaction. (B) Fever.

Among the 48 patients identified from two studies reporting adverse events, the overall rate was 0.042 (95% CI: 0.002–0.820). The leading two common side effects were hematological injection site reaction and fever. Injection site reaction is a common dermatological change characterized by pain, redness, and swelling at the site of injection. Injection site reaction is a typical local effect for LD‐IL‐2, occurring in 33.1% of patients with SLE (Figure 6A). Fever occurred in 14.4%, which was a common systemic effect of patients with SLE treated with LD‐IL‐2 (Figure 6B). Most of these adverse events were mild and recovered quickly after discontinuation. No serious adverse events were reported in all these studies, including Neuropsychiatric manifestations of SLE (NPSLE), Herpes zoster, and Pneumonia.

## 4 DISCUSSION

SLE is a classical systemic autoimmune disease in which the immune system attacks healthy cells and tissues throughout the body.^1^ The choice of therapy for SLE is primarily determined by how to maintain immune tolerance. Treg plays a crucial role in the modulation of immune responses and is known to play a critical role in autoimmune diseases. Many studies have described decreased proportions of Treg cells in patients with SLE.^21^ IL‐2 has been considered a proinflammation factor and has played an essential role in malignant tumors since it was discovered. It has been proven that IL‐2 is an indispensable cytokine required for the expansion and survival of Treg cells, which could maintain autoimmune tolerance. IL‐2 directed at Treg could have potential therapeutic value in SLE.^22^

Our meta‐analysis found that patients with SLE had a higher proportion of Treg after receiving LD‐IL‐2 compared with these patients before treatment. At the same time, there were no statistical differences in the balances of Th17 cells, Th1 cells, and Th2 cells before and after LD‐IL‐2 treatment. The proportions of NK cells were significantly decreased after LD‐IL‐2 injection. Besides, the proportions of CD4^+^ T cells were significantly increased after IL‐2 injection, while there were no statistical differences in the proportions of CD8^+^ T cells.

With a complete understanding of the function and biological characteristics of IL‐2, the pleiotropic function of IL‐2 has been uncovered. On the one hand, IL‐2 acts as a proinflammatory factor to promote autoimmune inflammatory responses such as Th1 and Th2.^23^, ^24^ On the other hand, it induces the differentiation of Treg and inhibits Th17 to maintain immune tolerance.^5^

**Figure 7:**
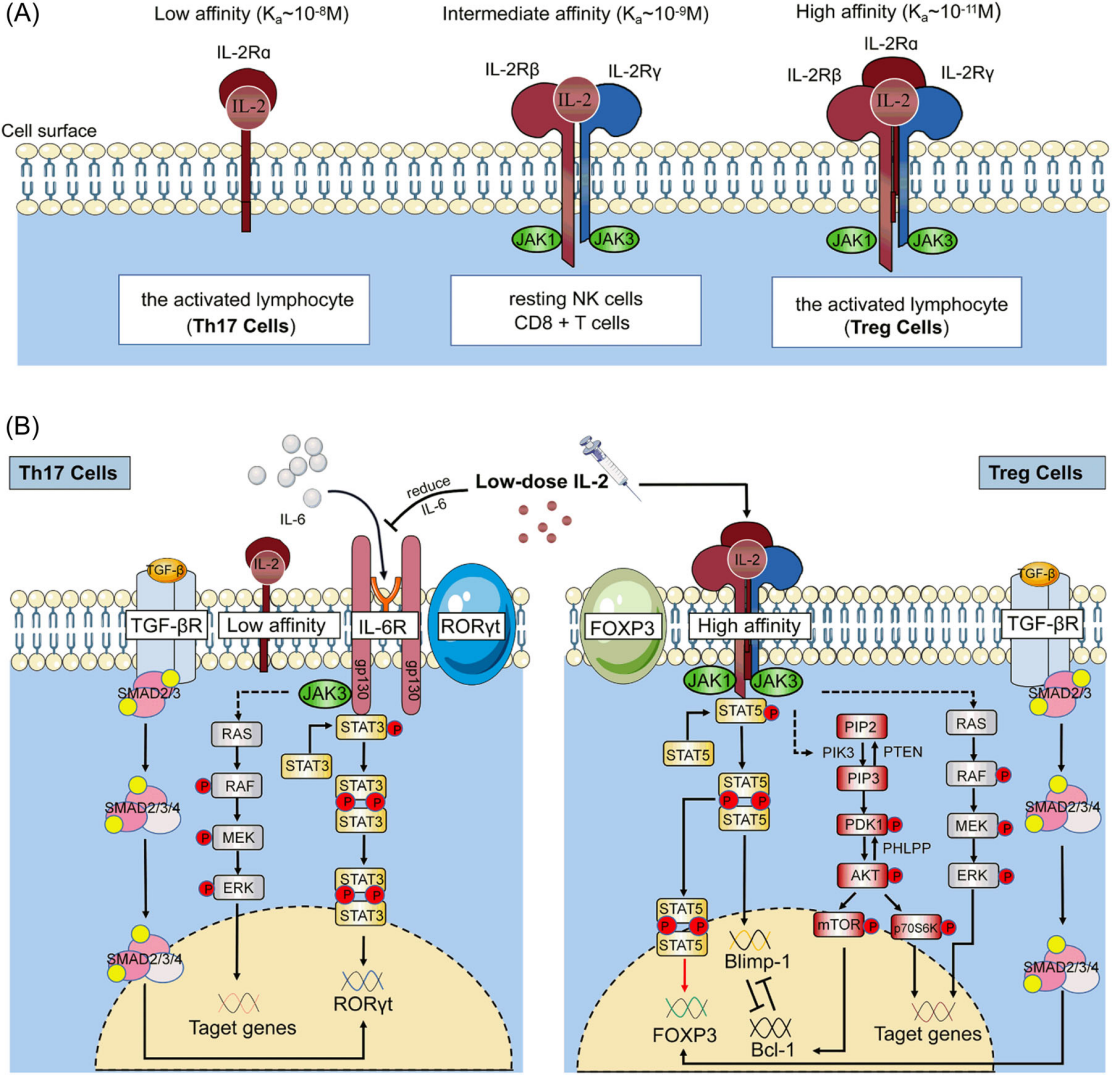
(A) The types of IL‐2R. (B) Low‐affinity IL‐2 receptor on Th17 cells hardly plays a role when the dose of IL‐2 is low, but Treg cells are quite the opposite. In Th17 cells, low‐dose interleukin‐2 (LD‐IL‐2) inhibits the JAK‐STAT3 signaling pathway by reducing IL‐6 to suppress RORγt expression, thus inhibiting Th17 differentiation. In Treg cells, IL‐2 promotes the phosphorylation of STAT5 through JAK1 and JAK3, leading to the expression of Foxp3 to promote the maturation and differentiation of Treg. Low‐dose IL‐2 also promoted Bcl‐1 expression and inhibited Blimp‐1 expression to promote the maturation and differentiation of Treg cells.

Treg and Th17 have the typical precursor cell but have different cell markers. Forkhead box P3 (Foxp3) is the marker of Treg, while RORγt is the marker of Th17.^25^, ^26^ Transforming growth factor‐β is an essential factor for inducing the expression of Foxp3 and RORγt, but STAT5 and STAT3 control the expression of Foxp3 and RORγt, respectively.^27^, ^28^, ^29^ Foxp3 is regulated by the JAK‐STAT5 signaling pathway by IL‐2.^27^ But RORγt is induced by the JAK‐STAT3 signaling pathway by IL‐6, and STAT3 can be suppressed by IL‐2.^30^ Therefore, the presence of IL‐2 promotes the differentiation of Treg via the STAT5 mechanism while inhibiting the differentiation of Th17 by preventing the activation of STAT3.^31^, ^32^ Besides, Treg expresses high‐affinity IL‐2 receptors (CD25), so it has superior efficacy in competing with other cells to bind IL‐2, which confers Treg dominance at LD‐IL‐2^5^, ^33^ (Figure 7).

Aside from its role in supporting Treg cells, the other main functions of IL‐2 are to support the proliferation of CD4^+^ T cells and the terminal differentiation of CD8^+^ T cells.^34^, ^35^, ^36^ Resting NK cells and CD8^+^ T cells, as important cells to exert cytotoxic effects to promote the immune response and kill tumor cells, express intermediate affinity receptors, which are slightly less sensitive to IL‐2 and can only be activated by high‐dose IL‐2.^37^, ^38^

NK cells modulate autoimmune diseases. A clear decrease in absolute and relative numbers of circulating NK cells was found in SLE, particularly in active disease. Active SLE was associated with CD56 NK cells.^39^ In sensitivity analysis, one of the main reasons for instability was differences among immune phenotypes counted in NK cells.

Proof‐of‐concept clinical trials have shown that at low doses, IL‐2 precisely and safely activates Treg cells in humans and improves autoimmune and alloimmune inflammatory conditions, but with limited clinical benefit and significant toxicity when used in high doses.^5^ However, we found in the clinic that different drug regimens for LD‐IL‐2 treatment will affect the treatment effect of patients. Currently, the common drug regimens in clinics are 0.5 million IU per day of IL‐2 injection for 5 days or 1 million IU of IL‐2 injection every other day for 2 weeks, followed by a 2‐week break. So, we conducted a subgroup analysis based on different dosages of LD‐IL‐2 and found that the proportions of Treg cells significantly increased after 0.5 million IU per day of IL‐2 injection for 5 days. Still, there were no statistical differences in the proportions of Treg cells after receiving 1 million IU of LD‐IL‐2 every other day. Special attention needs to be paid that IL‐2 has a short half‐life, which is connected to its small molecular weight. In line with these theoretical considerations, to maintain efficient IL‐2 bioavailability, the administration must be repeated at close intervals.^25^ In parallel, IL‐2 prefers to be administered subcutaneously rather than systemicly.^31^ Under the treatment of LD‐IL‐2 frequently (0.5 million IU per day), the increase of Treg cells is more significant than using IL‐2 at a little far interval (1 million IU every other day).

Our results show that treatment with LD‐IL‐2 was efficacious and tolerated in clinical patients with active SLE. The SRI‐4 response rates of patients with SLE who received 1 million IU of LD‐IL‐2 every other day for 2 weeks, followed by a 2‐week break, and measured after 12 weeks were 73.2%, and the SELENA‐SLEDAI scores of these patients were significantly decreased compared with before medication. In SLE patients, a significant decrease in the frequency of Tregs, and a significant increase in the frequency of Th17 cells in peripheral blood were demonstrated. Additionally, the ratio of Th17 to Treg cell frequency was found to be significantly increased along with increased SLEDAI scores. Thus, Th17/Treg is related to SLEDAI scores.^40^ Th17 cells in the active stage of the disease were significantly higher than those in the control group, while Th17 cells in the non‐active phase were not significantly different from those in the control group. IL‐2 affects disease activity in patients with SLE by reducing the Th17 cell level, decreasing SLEDAI.^31^ Jing et al. found that with the decrease of Treg cells, resolution of clinical manifestations included rash, oral ulceration, arthritis, vasculitis, alopecia and fever.^8^ Animal Experiments also mentioned that long treatment regimen of lD‐IL‐2 therapy alleviated renal histopathology and improved renal function.^41^ The study of Jing and colleagues revealed that the SRI‐4 response rate of the IL‐2 group was considerably higher than that of the placebo group, and more reductions in SELENA‐SLEDAI scores were observed in the IL‐2 group.^8^, ^11^ Besides, 54.8% of lupus nephritis patients had distinct clinical remission after the LD‐IL‐2 treatment. Jing et al. also found that the complete remission rate of lupus nephritis was significantly higher in the IL‐2 group than in the placebo group. Median proteinuria and urine erythrocytes were significantly reduced. Anti‐dsDNA was decreased while complements C3 and C4 were slightly increased. A significant expansion of Treg cells was also found.^42^ The two leading side effects of LD‐IL‐2 were hematological injection site reaction and fever. Most of these adverse events were mild and recovered quickly after discontinuation. No serious adverse events were reported in all included studies, including Neuropsychiatric manifestations of SLE (NPSLE), Herpes zoster, and Pneumonia.

Our study had several limitations. First, we did not consider the effects of disease duration, which may have contributed to the heterogeneity among studies. Second, the number of studies was inadequate for subgroup analysis, which may have biased our results. Third, because there are few RCT studies in the included studies, there are no other drug control groups or placebo groups in this meta‐analysis. Fourth, only published studies were included in the meta‐analysis, and unpublished articles were excluded; publication and study selection bias may still have affected our results. Therefore, further studies are needed to verify our results.

## 5 CONCLUSION

LD‐IL‐2 treatment was effective and well‐tolerated in patients with SLE. LD‐IL‐2 therapy induces the proliferation and functional recovery of Treg cells and inhibits the differentiation of Th17 and T follicular helper(Tfh) cells in patients with SLE, thereby reducing disease activity and restoring immune homeostasis. Most adverse events of LD‐IL‐2 were mild and recovered quickly after discontinuation. LD‐IL‐2 has been applied to treating autoimmune diseases.^35^, ^36^ Besides, our meta‐analysis provides evidence that injecting 0.5 million IU of IL‐2 daily can better induce the differentiation of Treg cells and maintain immune homeostasis than injecting 1 million IU every other day. LD‐IL‐2 therapy holds great promise for SLE. After IL‐2‐based treatment experimental stage for SLE, wide usage in clinical practice is promising. However, the best time point for LD‐IL‐2 therapy and the optimal dosage (0.5 million IU per day or 1.0 million IU every other day) need to be determined. Taken together, LD‐IL‐2 therapy has the potential to revolutionize SLE therapy but intensive and long‐term research is still needed to evaluate and improve this therapeutic approach.

## AUTHOR CONTRIBUTIONS

**Qin‐Yi Su** and **Sheng‐Xiao Zhang**: Designed study. **Sheng‐Xiao Zhang**, **Jing Luo**, and **Xin‐Miao Wang**: Searched literature. **Qin‐Yi Su**, **Jing Luo**, and **Jing‐Kai Di**: Selected data. **Sheng‐Xiao Zhang**, **Qin‐Yi Su**, and **Yi‐Xin Cao**: Analyzed data. **Sheng‐Xiao Zhang** and **Qin‐Yi Su**: Wrote manuscript. **Sheng‐Xiao Zhang** and **Qin‐Yi Su**: Contributed to manuscript revision, read, approved submitted version.

## CONFLICT OF INTEREST STATEMENT

The authors declare no conflict of interest.

## Supporting information

Supplementary information.

## Data Availability

The original contributions presented in the study are included in the article. Further inquiries can be directed to the corresponding authors.
