# Supplementary material for: Efficacy, safety and the lymphocyte subsets changes of low‐dose IL‐2 in patients with systemic lupus erythematosus: A systematic review and meta‐analysis
Source: Immun Inflamm Dis. 2024 Jan 24;12(1):e1165. doi: 10.1002/iid3.1165 (PMC10808945; doi:10.1002/iid3.1165)
Supplement: Supplementary file 1 — Supplementary information. [file IID3-12-e1165-s001.pdf]

Efficacy, safety, and the lymphocyte subsets changes of low-dose IL-2 in patients with systemic lupus erythematosus: A systematic review and meta-analysis

Qin-Yi Su[1,2,3], Jing Luo[1,2,3], Xin-Miao Wang[2,3], Jing-Kai Di[2,3], Yi-Xin Cao[2,3], Sheng-Xiao Zhang[1,2,3*]

1 Department of Rheumatology, The Second Hospital of Shanxi Medical University, Taiyuan, Shanxi, China.

2 Shanxi Provincial Key Laboratory of Rheumatism Immune Microecology, Taiyuan, Shanxi, China.

3 Key Laboratory of Cellular Physiology at Shanxi Medical University, Ministry of Education, Taiyuan, China.

Qin-Yi Su: qinyisu2019@163.com; Jing Luo: jing_luo132@163.com; Xin-Miao Wang: sxmuxm2333@a.sxmu.edu.cn; Jing-Kai Di: jingkaidi2000@163.com; Yi-Xin Cao: yixintransfer@163.com; Sheng-Xiao Zhang: shengxiao_zhang@163.com.

***Corresponding author:**

Sheng-Xiao Zhang: Postal address: 382, Wuyi Road, Taiyuan, Shanxi; Telephone numbers: 18734823329; E-mail address: zhangshengxiao1@sxmu.edu.cn;

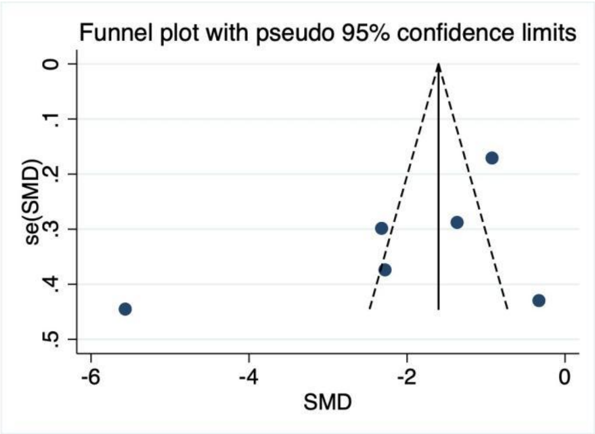

Figure S1: Funnel plot of the SELENA-SLEDAI scores.

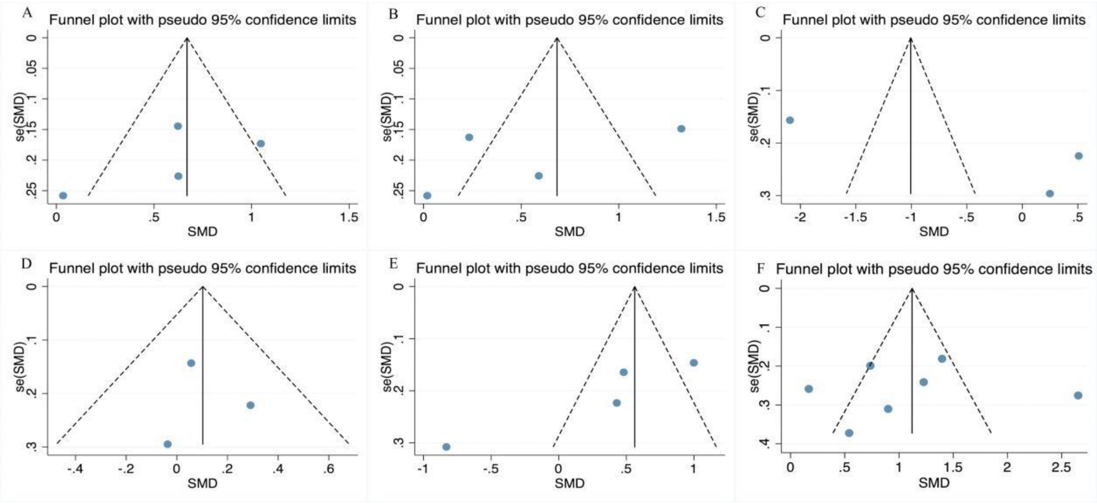

Figure S2: Funnel plot. (A)CD4+. (B)CD8+. (C)Th1. (D)Th2. (E)Th17. (F)Treg.

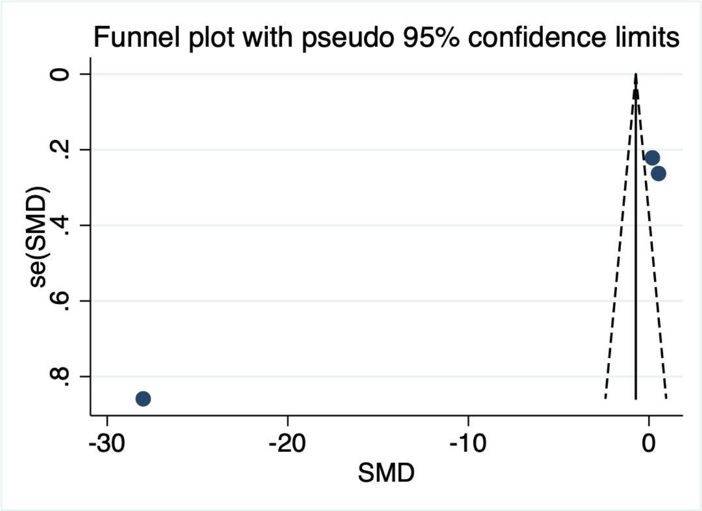

Figure S3: Funnel plot of the NK cells.

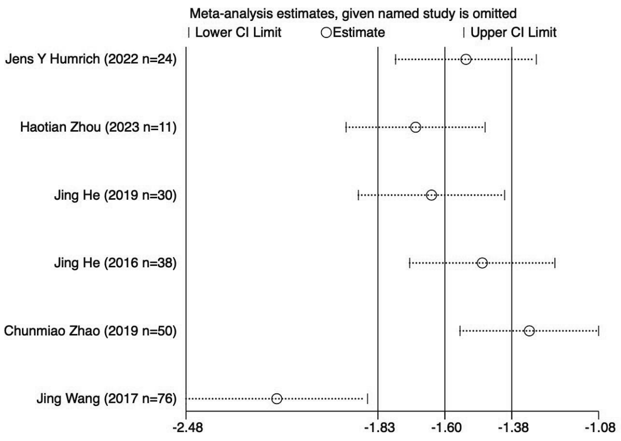

Figure S4: Sensitivity analysis of the SELENA-SLEDAI scores.

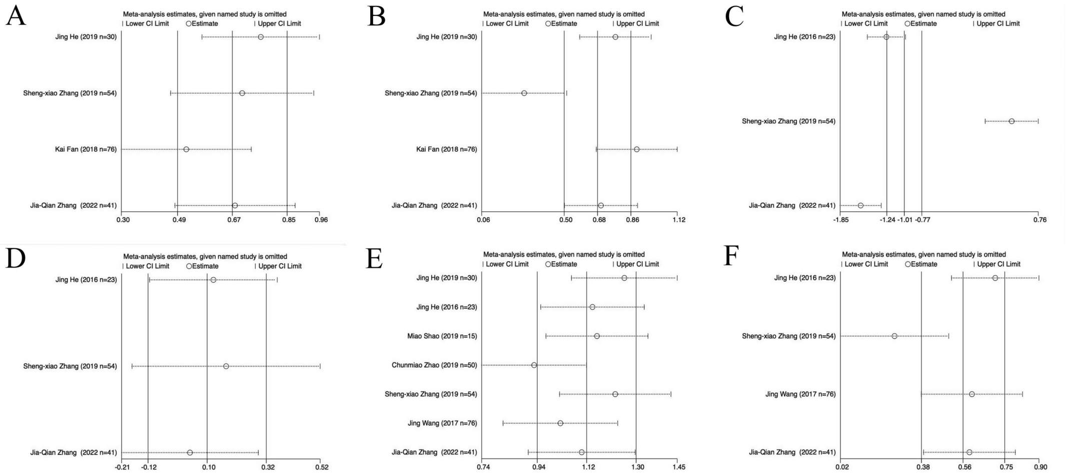

Figure S5: Sensitivity analysis. (A)CD4+. (B)CD8+. (C)Th1. (D)Th2. (E)Th17. (F)Treg.

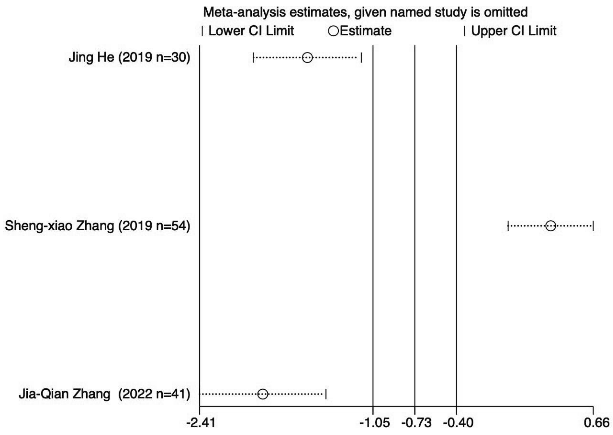

Figure S6: Sensitivity analysis of the NK cells.
